# Superficial acral fibromyxoma on the tip of the big toe: expression of CD10 and nestin

**DOI:** 10.1111/j.1468-3083.2007.02309.x

**Published:** 2008-02

**Authors:** N Misago, T Ohkawa, T Yanai, Y Narisawa

**Affiliations:** † Division of Dermatology, Saga University Saga, Japan; ‡ Division of Plastic and Reconstructive Surgery, Faculty of Medicine, Saga University Saga, Japan

## Editor

Superficial acral fibromyxoma is a rare, distinctive soft tissue benign neoplasm that has a predilection to develop on the hands and feet of adults. Except for the only rare location on the palm, superficial acral fibromyxoma usually involves the fingers and toes, and the big toe tends to be the most frequently affected site.^1^–^6^ Most cases of superficial acral fibromyxoma tend to occur in either the subungal or periungal regions, although only two rare cases occurring on the ventral surface of the digit have been previously described.^1^,^5^ We herein report a case of superficial acral fibromyxoma, which developed at the most common site (i.e. the big toe), but it occurred in a rare region of this toe (i.e. on the tip of the toe).

**fig. 1:**
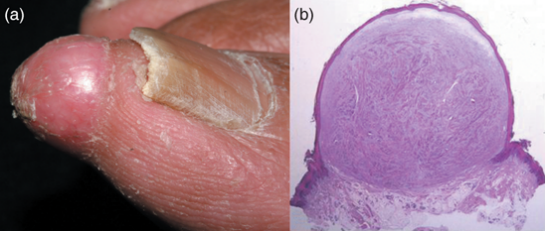
(a) A hemisphere-shaped, slightly reddish nodule on the tip of the patient's right big toe. (b) A scanning magnification, which was photographed directly to a slide using a special camera, showing a well-circumscribed, globularly projecting lesion to be located in the whole dermis while pushing against the subcutis (haematoxylin and eosin, ×1.5).

A 53-year-old healthy man presented with an asymptomatic, slowly growing nodule with a 3-year history on the tip of his right big toe. Examinations revealed a hemisphere-shaped, slightly reddish nodule, measuring 1.0 cm × 0.8 cm × 0.7 cm in size, on the tip of his right big toe (fig. 1a). Neither the involvement of the ungal region by the lesion nor any deformity of the nail plate was seen. Computed tomography scanning as well as plane radiographs revealed no involvement of the bone and no bone alterations. The nodule was completely excised, and a V-Y local advancement flap was also applied to the defect. Neither recurrence nor metastasis has been observed in the subsequent 1-year follow-up.

**fig. 2:**
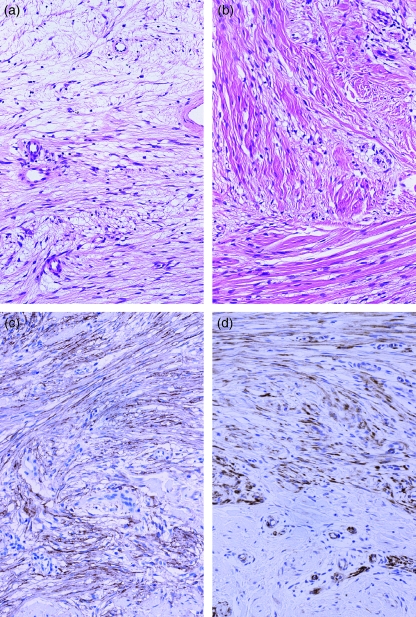
(a) The proliferated spindle and stellate cells with random and fascicular patterns in the myxoid stroma. (b) Those proliferated cells in the myxocollagenous stroma. (c) CD10 show positive staining in most of the neoplastic cells. (d) Nestin is positive for about 30% of the neoplastic cells mainly in the myxoid area. Note the labelling of nestin also in the endothelial cells of the capillaries and small vessels within the lesion. (haematoxylin and eosin: a, b ×50; Immunostain: c, d ×60).

Histopathologically, the excised nodule was a well-circumscribed, globularly projecting lesion, which was located in the whole dermis while pushing against the subcutis (fig. 1b). The lesion was composed of proliferated spindle and stellate cells with random, loose storiform, and fascicular patterns, which were embedded in myxoid or myxocollagenous stroma (fig. 2a,b). The proliferating cells showed no nuclear atypia, and strands of cells with wavy nuclei were sometimes seen. The accentuated vasculature and some inflammatory cells, composed of mast cells and lymphocytes, were also involved within the lesion. Alcian blue staining revealed abundant mucinous material within the stroma. Immunohistochemical study revealed that most of the neoplastic cells (> 80%) were positive for CD34, CD99, CD10, and Vimentin (fig. 2c), and they were negative for S-100 protein, epithelial membrane antigen. Factor XIIIa labelled only scattered dendritic cells within the lesion. Nestin was positive for about 30% of the neoplastic cells, mainly in myxoid area (fig. 2d). The histopathological and immunohistochemical features in the presented case closely corresponded to those of superficial acral fibromyxoma.

From a clinical point of view, the important points in superficial acral fibromyxoma are as follows: (i) a frequent deformity of the nail plate,^1^–^6^ (ii) a usual need for the removal of the nail plate during surgical procedures,^3^,^6^ and (iii) a rare deformity of the bone,^1^,^6^ because most cases of this condition affect either the subungal or periungal regions. Owing to its rare location (i.e. the tip of the toe), the case presented herein showed no deformity of either the nail plate or bone, and this lesion could therefore be completely excised without the need to remove the nail plate.

The histogenesis of superficial acral fibromyxoma is still unclear. Within the skin, the expression of CD10 has been reported in normal mesenchymal cells in the nail unit.^7^ Recent investigations have revealed that multipotent precursors isolated from the dermis, which can differentiate into both neural and mesodermal progeny, also express nestin.^8^–^10^ Therefore, the presented case may suggest that the neoplastic cells in this condition are related to the mesenchymal cells in the nail unit (onychoblasts) and show dedifferentiation into or include multipotent dermal stem cells.
